# A genome-wide association study identifies four novel susceptibility loci underlying inguinal hernia

**DOI:** 10.1038/ncomms10130

**Published:** 2015-12-21

**Authors:** Eric Jorgenson, Nadja Makki, Ling Shen, David C. Chen, Chao Tian, Walter L. Eckalbar, David Hinds, Nadav Ahituv, Andrew Avins

**Affiliations:** 1 Kaiser Permanente Northern California, Division of Research, Oakland, California 94612, USA; 2 Department of Bioengineering and Therapeutic Sciences, UCSF, San Francisco, California 94158, USA; 3 Institute for Human Genetics, UCSF, San Francisco, California 94158, USA; 4 Lichtenstein Amid Hernia Clinic, David Geffen School of Medicine at University of California Los Angeles, Los Angeles, California 90095, USA; 5 23andMe Inc. 899 W. Evelyn Avenue, Mountain View, California 94041, USA

## Abstract

Inguinal hernia repair is one of the most commonly performed operations in the world, yet little is known about the genetic mechanisms that predispose individuals to develop inguinal hernias. We perform a genome-wide association analysis of surgically confirmed inguinal hernias in 72,805 subjects (5,295 cases and 67,510 controls) and confirm top associations in an independent cohort of 92,444 subjects with self-reported hernia repair surgeries (9,701 cases and 82,743 controls). We identify four novel inguinal hernia susceptibility loci in the regions of *EFEMP1*, *WT1*, *EBF2* and *ADAMTS6*. Moreover, we observe expression of all four genes in mouse connective tissue and network analyses show an important role for two of these genes (*EFEMP1* and *WT1*) in connective tissue maintenance/homoeostasis. Our findings provide insight into the aetiology of hernia development and highlight genetic pathways for studies of hernia development and its treatment.

## Abstract (web summary)

Inguinal hernia has high lifetime prevalence, especially in men. This genome-wide association study identifies 4 loci to be associated with inguinal hernia, and shows expression of nearby genes in mouse connective tissues.

## Graphical abstract

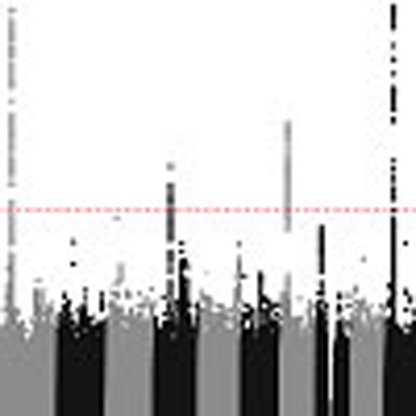

Inguinal hernias are amongst the most frequently diagnosed conditions in clinical practice and have a lifetime prevalence in the range of 20–27% in men and 3–6% in women^1^,^2^. They can be classified as either direct, which occur though an acquired weakness in the transversalis fascia, connective tissue that comprises the floor of the inguinal canal, or indirect, in which abdominal contents protrude through a congenital defect in the inguinal ring via a patent processus vaginalis. Inguinal hernia repair is one of the most common surgical procedures, with more than 750,000 performed annually in the United States^3^,^4^, and is associated with substantial costs^5^,^6^. Inguinal hernias can lead to serious medical morbidity such as bowel incarceration and strangulation, and emergency hernia surgery to treat these conditions is associated with a substantial mortality risk^7^,^8^. A subset of patients experience hernia recurrence after surgery and chronic pain affects over 6% of patients^9^, highlighting the need for a better understanding of hernia aetiology, which could, in turn, lead to new approaches to therapy and improved treatment outcomes.

Several risk factors underlying the development of inguinal hernia in adults have been identified, including male sex^2^,^7^, older age^2^, chronic obstructive pulmonary disease^10^, lower body mass index^11^ and family history^2^,^10^,^12^. The risk of inguinal hernia is increased among first-degree relatives of individuals with a history of inguinal hernia, suggesting that there likely exist identifiable genetic risk factors responsible for many inguinal hernias^12^,^13^. In addition, individuals with certain genetic syndromes, including cutis laxa^14^, Marfan syndrome^15^ and Ehlers-Danlos syndrome^16^, have a greater risk of developing inguinal hernias. To date, only a small number of candidate genes have been investigated^17^,^18^,^19^,^20^,^21^. As a result, little is currently known about the specific genes that play a role in the pathophysiology of inguinal hernia.

To address this question, we conduct the first large-scale genome-wide association study (GWAS) of surgically confirmed inguinal hernia. We utilize information from participants in the Genetic Epidemiology Research in Adult Health and Aging (GERA) cohort (*n*=110,266), nested in the Kaiser Permanente integrated health plan in Northern California (KPNC). We confirm top associations in a large independent sample of research participants with self-reported hernia repair surgery from 23andMe (*n*=92,444). We then examine patterns of expression of genes in the associated regions in mouse connective tissue equivalent to human transversalis fascia and find that all four genes are expressed in this tissue, supporting their role in hernia development.

## Results

Using information extracted from KPNC electronic health records (EHR), we identified hernia cases and controls among non-Hispanic white GERA participants and validated a subset of cases through chart review. In total, we identified 5,295 surgically confirmed inguinal hernia cases with male predominance (90.2%) and 67,510 controls with no known surgical or medical history of inguinal or other abdominal hernia in the GERA discovery cohort (Supplementary Table S1). Hernia repair discharge procedure codes indicated that 2,335 inguinal hernia cases had direct inguinal hernia repairs and 2,647 had indirect inguinal hernia repairs. We reviewed 230 patient charts to validate the accuracy of inguinal hernia diagnoses, and, of those, 228 (99.1%, 95% confidence interval (CI): 96.9–99.9%) were confirmed to be designated correctly as inguinal hernias of any type. Of the 118 charts reviewed specifically for accuracy of the diagnosis of direct inguinal hernia, 113 (95.7%, 95% CI: 90.3–98.6%) were found to be correctly identified. For indirect inguinal hernias, 110 of 112 chart diagnoses were found to be supported by the clinical data (98.2%, 95% CI: 93.7–99.8%). Thus, the positive predictive value of our algorithm for identifying hernia cases, as well as hernia type, was very high in this sample.

### Genetic association analysis of inguinal hernia

**Figure 1:**
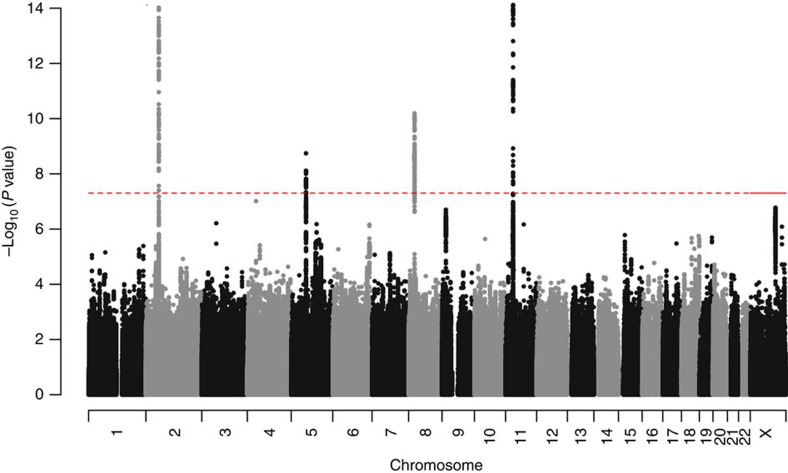
Manhattan plot of GWAS findings in the GERA discovery cohort. Four novel inguinal hernia risk loci with genome-wide significant associations were identified in the regions of *EFEMP1* (chromosome 2), *ADAMTS6* (chromosome 5), *EBF2* (chromosome 8) and *WT1* (chromosome 11). The dotted red line represents a significance threshold of *P*=5.0 × 10^-8^.

We conducted a sex-stratified GWAS analysis of inguinal hernia in the GERA cohort, adjusting for age and the first 10 ancestry principal components. The genomic control *λ* values were 1.022 for the analysis of men and 1.021 for the analysis of women. We identified four loci that exceeded genome-wide significance (*P*<5 × 10^−8^) in the regions of *EFEMP1* (rs2009262, odds ratio (OR)=1.23, *P*=3.66 × 10^−15^), *WT1* (rs3809060, OR=1.18, *P*=4.69 × 10^−14^), *EBF2* (rs6991952, OR=1.14, *P*=1.17 × 10^−10^) and *ADAMTS6* (rs370763, OR=1.14, *P*=9.70 × 10^−9^) in the discovery cohort (Fig. 1, Table 1 and Supplementary Fig. 1). We confirmed these associations in 9,701 cases and 82,743 controls who were research participants from the 23andMe cohort with self-reported information on history of hernia repair surgery (Supplementary Table S2). In this replication cohort, we observed significant associations of all four single-nucleotide polymorphisms (SNPs; rs2009262, OR=1.10, *P*=3.65 × 10^−6^, rs3809060, OR=1.07, *P*=1.69 × 10^−4^, rs6991952, OR=1.08, *P*=2.04 × 10^−6^ and rs370763, OR=1.06, *P*=3.02 × 10^−3^; Table 1).

Each of these inguinal hernia risk genes has a plausible biological and pathophysiologic role in the development of hernias, which is known to have metabolic aetiology related to collagen subtype and maturation, elastin and matrix metalloproteinases, in addition to congenital and acquired factors. *EFEMP1* knockout mice develop both direct and indirect inguinal hernias, have reduced elastic fibres in fascia and display signs of early aging^22^. Nonsynonymous variants in *WT1* have been identified in patients with Denys–Drash syndrome and Meachem syndrome with congenital diaphragmatic hernia^23^,^24^,^25^. An antisense morpholino knockdown study of *EBF2* resulted in defects in muscle development in *Xenopus*^26^. *ADAMTS6* is a member of a gene family that encode proteases that convert procollagen to collagen^27^. Mutations in the gene family member *ADAMTS2* have been associated with Ehlers–Danlos syndrome with congenital umbilical hernia^28^.

To determine whether there were additional inguinal hernia risk alleles in the four inguinal hernia susceptibility loci, we repeated the GWA analysis in the GERA sample conditioning on the top associated SNPs at each of the four loci. We did not observe any other SNPs that were significantly associated with inguinal hernia in the conditional analysis. We then estimated the point prevalence of surgically confirmed inguinal hernia among non-Hispanic white KPNC members who were at least 50 years of age as of June 2013, which was 9.2% in men and 0.3% in women. These estimates are consistent with the lifetime prevalence of inguinal hernias previously reported in the literature, 27% for men, 6% for women, but lower due to the more stringent case definition and shorter observation time. Using both the point and lifetime prevalence estimates to provide a range, the four top SNPs explained 1.0–1.4% of the variation in the risk of inguinal hernia in men and 1.3–2.8% in women in our discovery sample. The narrow-sense heritability explained by common SNPs (minor allele frequency >5%) ranged from 13.2 to 18.3% in men and 20.8 to 25.5% in women, suggesting that additional inguinal hernia susceptibility loci remain to be discovered.

### Direct and indirect inguinal hernia

Inguinal hernias can be classified as direct, in which the abdominal contents herniate through the floor of the inguinal canal due to an acquired weakness in the transversalis fascia, or indirect, in which abdominal contents protrude through a congenital defect in the inguinal ring via enlargement of a patent processus vaginalis. We analysed the four inguinal hernia risk SNPs in GERA subjects with direct and indirect hernias separately to determine whether any of them predisposed subjects to a specific subtype of inguinal hernia. The ORs observed for direct inguinal hernia were slightly stronger for three of the four top SNPs in men than for indirect inguinal hernia (Table 2). In women, for whom there were fewer subjects with inguinal hernias (*N*=549), only rs2009262 and rs3809060 were nominally associated with direct or indirect inguinal hernia (*P*<0.05), and both displayed larger effects for indirect compared with direct inguinal hernia.

We then examined the association of SNPs in the region (±250 kb) of the four hernia susceptibility loci with direct and indirect hernia in men. The four top SNPs associated with inguinal hernia were also the most strongly associated SNPs with indirect inguinal hernia, but for three of the four loci, other SNPs in the region were more strongly associated with direct inguinal hernia, specifically rs11899888 (instead of rs2009262) in *EFEMP1*, rs12520760 (instead of rs370763) in *ADAMTS6* and rs10746560 (instead of rs6991952) in *EBF2* (Supplementary Fig. 2). This indicates that multiple variants within these risk loci may underlie the different subtypes of inguinal hernia.

To determine whether specific biological pathways or functions play a role in inguinal hernia development, we conducted a gene set enrichment analysis of our discovery cohort results using the program Meta-Analysis Gene-set Enrichment of variant Associations (MAGENTA)^29^. We identified four gene sets at a false discovery rate (FDR) <0.05: Jak Stat signalling, leukocyte extravasation signalling, actin cytoskeleton signalling and glycosaminoglycan biosynthesis chondroitin sulfate (Supplementary Table 3). We then used RegulomeDB to investigate the potential for SNPs in the identified inguinal hernia risk loci to influence the binding of transcription factors^30^. We identified 14 SNPs in the four regions that were classified as likely to affect transcription factor binding (Supplementary Table 4).

### Expression of inguinal hernia risk genes

**Figure 2:**
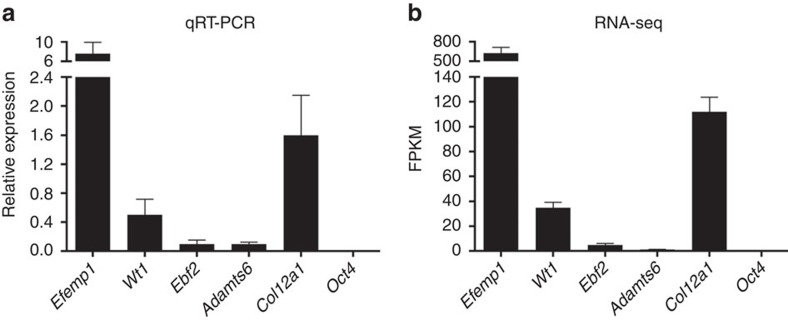
Expression analysis of *Efemp1*, *Wt1*, *Ebf2* and *Adamts6* by qRT-PCR (a) and RNA-seq (b). *Efemp1* is expressed at a high level, *Wt1* at a moderate level and *Ebf2* and *Adamts6* at low levels in mouse connective tissue compared to a connective tissue expressed gene *Col12a1* (positive control) and *Oct4* that is not expressed in this tissue (negative control). Data are represented as mean±s.d. for the qRT-PCR and ±s.e.m. for the RNA-seq (*n*=12). For qRT-PCR three samples were analysed in three replicates of each reaction and relative expression levels calculated by the ΔC_T_ method. For RNA-seq, three replicates were analysed and normalized gene expression values, FPKM, were obtained for each replicate using Cufflinks2.

Using quantitative real-time PCR (qRT-PCR) and RNA sequencing (RNA-seq), we examined mRNA levels of the four genes in mouse connective tissue equivalent to human transversalis fascia (see Methods section). qRT-PCR found *Efemp1* to be expressed at a high level, *Wt1* at a moderate level and *Ebf2* and *Adamts6* at low levels compared with a control connective tissue expressed gene (*Col12a1*; Fig. 2a). Our RNA-seq analysis showed comparable fragments per kilobase per million reads (FPKM) values, with all four genes correlating well with the relative expression levels determined by qRT-PCR (Fig. 2b). Combined, our results show that all four genes are expressed in connective tissue and could have a functional role in this tissue.

**Figure 3:**
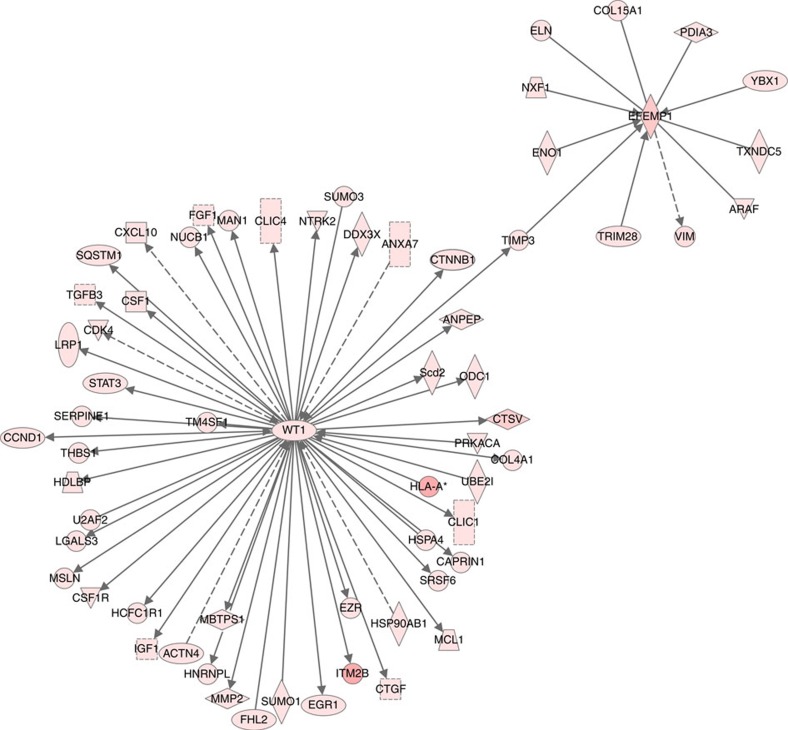
Ingenuity Pathway Analysis outlines potential regulatory networks around EFEMP1 and WT1. Network Analysis for EFEMP1 and WT1 was carried out using the RNA-seq FPKM>30 gene list (see Methods section). WT1 regulates many extracellular matrix genes, including MMP2 (matrix metalloproteinase-2) and CTGF (connective tissue growth factor). EFEMP1 directly interacts with ELASTIN, a component of elastic fibres in the ECM. TIMP3 (tissue inhibitor of metalloproteinase-3), which is activated by WT1 and interacts with EFEMP1 and was found to connect between the two networks.

We next set out to characterize the gene regulatory networks associated with these genes. We carried out Causal Network Analysis on the highest expressing genes from our RNA-seq list (see Methods section) using the Ingenuity Pathway Analysis software (IPA, Qiagen). Since *Ebf2* and *Adamts6* were expressed at low levels, we only characterized interactions for *Efemp1* and *Wt1*. We identified many interesting interactors for EFEMP1 including ELASTIN, a component of elastic fibres and COLLAGEN15A1, a component of collagen fibres (Fig. 3). The WT1 network contained many extracellular matrix (ECM) proteins. These included MMP2 (matrix metalloproteinase-2), CTGF (connective tissue growth factor) and THBS1 (thrombospondin-1), all proteins known to play a role in connective tissue remodelling and homoeostasis. One common protein of interest between the two networks is TIMP3 (tissue inhibitor of metalloproteinase-3), which inhibits matrix metalloproteinases that degrade collagen and elastin. TIMP3 interacts with EFEMP1 and is thought to be activated by WT1. Changes in the expression levels of TIMP3 could shift the intricate balance between ECM degrading and protecting enzymes and may thus perturb connective tissue homoeostasis. Combined, our IPA analysis suggests that EFEMP1 and WT1 play a role in connective tissue maintenance/homoeostasis through their action on collagen and/or elastin.

## Discussion

We identified four novel inguinal hernia genetic susceptibility loci near the genes *WT1*, *EFEMP1*, *EBF2* and *ADAMTS6*, and confirmed those associations in an independent cohort. All four loci appear to be associated with both direct and indirect inguinal hernias. Each of these four genes is expressed in mouse connective tissue, with the expression of *EFEMP1* being particularly high. Our IPA analysis suggests that WT1 and EFEMP1 might play a role in connective tissue maintenance/homoeostasis through their action on ECM enzymes including matrix metalloproteinases that degrade collagen and elastin fibres.

Dysregulation of collagen homoeostasis is thought to play an important role in the development of inguinal hernias^31^. Collagen is the main structural protein of the abdominal fascia, and undergoes a continuous process of synthesis and degradation^32^. Transversalis fascia samples from patients with indirect inguinal hernias were found to have lower levels of collagen compared with cadaver controls and showed a decreased ratio of type I to type III collagen^33^. The alteration of this ratio appears to be driven by greater expression of type III collagen mRNA in patients with inguinal hernias compared with controls^34^. In addition, an imbalance in the activity of collagen degrading matrix metalloproteinases and their inhibitors (MMPs and TIMPs) has been reported in fibroblasts of patients with inguinal hernias^35^. WT1 has been shown to inhibit MMP2 (ref. 36) and activate TIMP3 (ref. 37), which in turn inhibits MMPs. EFEMP1 interacts with TIMP3 and might thus augment the inhibitory role of WT1 on MMPs^38^. In addition, *ADAMTS* family members are matrix metalloproteinases that convert procollagen to collagen^27^. The association of genetic variants near *ADAMTS6* supports the hypothesis that collagen dysregulation can influence the development of inguinal hernias. A GWAS of central corneal thickness (CCT) also identified the *ADAMTS6* locus, along with an association with the collagen gene *COL5A1* (ref. 39), suggesting that *ADAMTS6* may influence collagen homoeostasis in multiple tissues and disorders.

Elastin is also a key component of transversalis fascia that complements the role of collagen by providing elasticity, which allows for the tissue to stretch and return to its original form. Mutations in the human elastin gene, *ELN*, cause cutis laxa^40^, which has been associated with an increased risk of inguinal hernias^14^ and supravalvular aortic stenosis^41^. In connective tissue, the integration of elastin to the microfibril scaffold is guided by fibulins^42^; *EFEMP1* is a member of the fibulin gene family, and the EFEMP1 protein binds tropoelastin, the building block of the elastin protein^43^. *EFEMP1* knockout mice have reduced elastic fibres in fascia and develop direct and indirect inguinal hernias^22^. Variants in the *EFEMP1* locus have also been associated with a number of conditions and functional changes, including differences in forced vital capacity, a measure of lung function^44^. This shared association suggests that alterations in elastin maintenance may contribute to the development of both chronic obstructive pulmonary disease and inguinal hernia and may be the mechanism through which chronic obstructive pulmonary disease increases the risk of inguinal hernias. These alterations in elastin and connective tissues may act more generally to affect the risk of disorders of other elastic tissues, such as abdominal aortic aneurysm, for which inguinal hernia patients are at an increased risk^32^,^45^.

While this is the first study to identify inguinal hernia susceptibility loci, previous GWASs have identified these regions as influencing a number of human phenotypes, supporting a functional role for variation in inguinal hernia loci in human traits and diseases. *WT1*, so named for causing Wilm's tumour^46^, has also been associated with tuberculosis^47^. Variants in the *EFEMP1* locus have been associated with height^48^ and forced vital capacity^44^, and its epigenetic silencing has been associated with multiple cancer types^49^,^50^. *EBF2* has been associated with prostate cancer, though the variants identified were located proximal to those identified here^51^. SNPs in the *ADAMTS6* region are associated with differences in CCT, an anthropomorphic measure of the eye, but not conditions associated with CCT, including keratoconus or primary open-angle glaucoma^39^. A second study also found suggestive evidence for association of this locus with osteosarcoma^52^. The pleiotropic effect of the loci identified in this study suggests a potential shared aetiology between inguinal hernia risk and cancer, lung function and anthropomorphic traits. Given previous observational associations between inguinal hernia risk and body mass index and other connective tissue disorders, examining potential shared effects of genetic variation underlying these disorders may provide additional insight into hernia development.

Although these lines of evidence provide support for the role of these four genes in hernia development, further experiments are needed to demonstrate a causal role for these genes and specific SNPs in the gene regions. These experiments include examining epigenetic features by performing ChIP-seq (chromatin immunoprecipitation followed by deep sequencing) on fascia connective tissue and identifying SNPs that reside in putative gene regulatory regions that are also in linkage disequilibrium with SNPs associated with inguinal hernia risk. Complementary to this, differential enhancer assays can be carried out in human fibroblast cell lines to compare enhancer activity of the reference allele and the potential risk allele. Genome editing techniques, such as CRISPR/Cas9, can also be used to delete the regulatory region or to replace the reference allele with the risk allele, allowing for a more complete understanding of mechanisms through which the risk alleles act to influence the development of inguinal hernias.

The incidence of hernia susceptibility in humans peaks at birth and late adulthood^2^. It is possible, and perhaps likely, that factors influencing both the development of fascia and their maintenance affect inguinal hernia susceptibility. Our discovery sample focused on surgically confirmed adult-onset inguinal hernia, with an average age of 66.2 years at diagnosis. It is unclear how the inguinal hernia risk loci identified here influence the risk of childhood-onset hernias, which are always of the indirect type and related to congenital persistence of the processus vaginalis. Furthermore, we confirmed our findings in subjects with self-reported hernia repair surgery, which likely represents a mix of hernia subtypes, with inguinal hernias being the most common. First-degree relatives of inguinal hernia patients have a greater risk of femoral, incisional, epigastric and umbilical hernias^16^, indicating a common metabolic aetiology and a shared genetic basis across different hernia subtypes, which may, in part, explain why we observe similar signals across the two cohorts. Future research should examine how the loci identified here contribute to the risk of other types of hernias and the extent to which the mechanisms underlying inguinal hernia development are common to other hernia subtypes and other connective tissue disorders.

In conclusion, our study identified four novel loci underlying the risk of adult-onset inguinal hernia. Our findings suggest a role for the regulation of both collagen homoeostasis and elastin maintenance in the development of inguinal hernias, which appear to also influence anthropomorphic traits, the risk of cancer and lung function. Further research into the precise mechanisms through which these loci act may improve our understanding of hernia formation and point the way to more effective preventative, operative and non-surgical treatments of this common disorder.

## Methods

### Setting

KPNC is an integrated healthcare delivery organization, which has an active membership of 3.5 million people. It is the largest healthcare provider in Northern California. Approximately, one third of the Northern California population is enroled in the KPNC health plan. Comparisons with the general population have shown that the membership is a representative of the population of Northern California, with the exception of extremes of the socioeconomic spectrum^53^. In 1995, KPNC instituted a comprehensive EHR system, which records physician diagnoses, prescriptions and lab results from all inpatient and outpatient encounters. KPNC has high membership retention, with over 90% of those over age 65, and 66% of all active members as of June 2012, having five or more years of retrospective membership.

### The GERA cohort

The GERA cohort is comprised of 110,266 adult men and women members of the KPNC Medical Care Plan. It is a component of the KPNC Research Program on Genes, Environment and Health. The detailed description of the cohort and study design can be found in dbGaP (Study Accession: phs000674.v1.p1). Briefly, participants were enroled through participation in a mailed survey of all adult members of KPNC (∼1.9 million) conducted in 2007. A total of 435,983 members completed the 5-page survey, which included information on demographic factors, behaviours and self-reported health. Beginning in July 2008, respondents to the survey were asked to sign and return a consent form authorizing use of biospecimens, survey data and data from participants' EHR for use in studies of genetic and environmental influences on health and disease. Respondents who completed consent forms were mailed (Oragene) saliva collection kits. A total of 110,266 participant samples were selected for genome-wide genotyping and telomere length measurement to ensure that at least 100,000 were successfully assayed (102,998 samples passed genotyping quality control). The average age of the participants at the time of sample collection was 62.9 years old (s.d.=13.8 years); 69,987 participants (63%) were aged 60 years and older, and over 12,000 were aged 80 years and older. The sample is ethnically diverse, generally well-educated, with above average incomes. Length of membership in KPNC averaged 23.5 years, indicating the stability of KPNC membership and the length of medical history that is recorded for cohort members. All study procedures were approved by the Institutional Review Board of the Kaiser Foundation Research Institute.

### 23andMe cohort

Study participants in the replication cohort were drawn from the customer base of 23andMe, which has been previously described in detail^54^,^55^. All individuals provided informed consent and answered surveys online according to the 23andMe human subjects protocol, which was reviewed and approved by Ethical and Independent Review Services, a private institutional review board (http://www.eandireview.com).

### Phenotype definition

Hernia cases in the GERA cohort were identified from clinical diagnoses and surgical procedures captured in the EHR system. Hernia repair surgeries were typically associated with pre-operative and post-operative diagnoses and a detailed operative report; hernias found or repaired among inpatients also resulted in hospital discharge diagnoses. The operative reports were reviewed by hospital coders so that the corrected discharge diagnosis and procedure codes were assigned. These procedure codes usually indicated whether an inguinal hernia repair was for a direct or indirect hernia based on information contained in the operative report. These hernia diagnoses and surgical operations were recorded in the EHR system as International Classification of Disease, Ninth Revision (ICD9) diagnosis and procedure codes as well as Common Procedure Terminology, Fourth Edition (CPT4) codes. For this study, we required that inguinal hernia cases must have had a hospital discharge diagnosis of inguinal hernia or an inguinal repair surgery with a post-operative diagnosis of inguinal hernia. Diagnosis and procedure codes are listed in Supplementary Table 5.

To establish the validity of our case definition, chart review was conducted by a board-certified internist (A.A.) with the assistance of a board-certified general surgeon with special expertise in hernia repair (D.C.C.). The chart review consisted of reading the full, original narrative operative report and determining the performing surgeon's post-operative diagnosis and ensuring it was consistent with the procedure as described in the text of the operative report.

In the 23andMe cohort, case–control status was determined using the answer to one question from the ‘Your Medical History' Survey: ‘Have you ever had any of the following gastrointestinal surgeries (Hernia repair)?'

### Genotyping, quality control, imputation and genetic ancestry

*Genetic Epidemiology Research in Adult Health and Aging*. DNA was extracted from Oragene kits at KPNC and genotyped at UCSF using the Affymetrix Axiom EUR arrays as previously described (dbGaP Study Accession: phs000674.v1.p1). Briefly, samples with dish quality control (DQC) <0.82 or initial genotype call rate <0.97 were excluded, resulting in a total of 83,285 individuals of Europeans ancestry in our analysis^56^. To improve genotype calls, SNPs were re-called within packages of plates assayed under similar conditions (array type, reagent, hibernation time and DNA concentration). SNPs were removed if either package call rate or overall call rate (across packages) was below 90%. Additional SNP exclusion criteria were (1) large allele frequency variance across packages—defined as the ratio of overall variance of the SNP allele frequency across packages to the sample SNP heterozygosity (total sample variance; <31); (2) large allele frequency differences between males and females (>0.15) for autosomal SNPs; and (3) poor concordance among duplicates (<0.24).

Following the quality control steps, genotypes were pre-phased with Shape-IT v2.r727 (ref. 57) then imputed to a cosmopolitan reference panel consisting of all of the individuals from the 1000 Genomes Project^58^ (March 2012 release) using IMPUTE2 v2.3.0 and standard procedures^59^. The info-metric from IMPUTE2 is a quality measure, estimating the correlation (*r*^2^) of the imputed genotype to the true genotype. Herein, we reported SNP associations with info >0.8 and minor allele frequency >0.05. The most strongly associated SNPs at the four loci reported here had high imputation *r*^2^ values (rs2009262: 0.996; rs3809060: 0.967; rs6991952, which was genotyped directly: 1.000; and rs370763: 0.961) As a result of these quality control steps, we assessed a total of 6,161,781 SNPs in the GWAS analyses.

EIGENSTRAT (http://genepath.med.harvard.edu/~reich/EIGENSTRAT.htm) was used to compute eigenvectors with 41,228 high-quality SNPs that were common amongst all arrays and the human genome diversity project (dbGaP Study Accession: phs000674.v1.p1)^60^. Since the principal component analysis was computationally intensive, it was run on a large set of individuals (*N*=20,000) with the remaining individuals projected into the same space. These principal components were used in the GWAS to adjust for genetic ancestry.

*23andMe*. DNA extraction and genotyping were performed on saliva samples by National Genetics Institute, a CLIA-licensed clinical laboratory and a subsidiary of Laboratory Corporation of America. Samples were genotyped on one of four genotyping platforms. The V1 and V2 platforms were based on the Illumina HumanHap550+ BeadChip, including about 25,000 custom SNPs selected by 23andMe, with a total of about 560,000 SNPs. The V3 platform was based on the Illumina OmniExpress+ BeadChip, with custom content to improve the overlap with the V2 array, with a total of about 950,000 SNPs. The V4 platform in current use is a fully custom array, including a lower redundancy subset of V2 and V3 SNPs with additional coverage of lower-frequency coding variation, and about 570,000 SNPs. Samples that failed to reach 98.5% call rate were reanalyzed. Individuals whose analyses failed repeatedly were re-contacted by 23andMe customer service to provide additional samples.

The subjects to be analysed were restricted to a set of individuals who have >97% European ancestry, as determined through an analysis of local ancestry^61^. Briefly, the algorithm first partitions phased genomic data into short windows of about 100 SNPs. Within each window, a support vector machine is used to classify individual haplotypes into one of 31 reference populations. The support vector machine classifications are then fed into a hidden Markov model that accounts for switch errors and incorrect assignments, and gives probabilities for each reference population in each window. Finally, simulated admixed individuals are used to recalibrate the hidden Markov model probabilities so that the reported assignments are consistent with the simulated admixture proportions. The reference population data is derived from public data sets (the Human Genome Diversity Project, HapMap, and 1000 Genomes), as well as 23andMe customers who have reported having four grandparents from the same country.

A maximal set of unrelated individuals was chosen for each analysis using a segmental identity-by-descent estimation algorithm^62^. Individuals were defined as related if they shared more than 700 cM identity-by-descent, including regions where the two individuals share either one or both genomic segments identical-by-descent. This level of relatedness (roughly 20% of the genome) corresponds approximately to the minimal expected sharing between first cousins in an outbred population.

Participant genotype data were imputed against the September 2013 release of 1000 Genomes Phase1 reference haplotypes, phased with Shape-IT^57^,^63^. Data for each genotyping platform was phased and imputed separately. Phasing was conducted using a phasing tool, Finch, developed at 23andMe, which implements the Beagle haplotype graph-based phasing algorithm^64^, modified to separate the haplotype graph construction and phasing steps. Finch extends the Beagle model to accommodate genotyping error and recombination, to handle cases where there are no consistent paths through the haplotype graph for the individual being phased. Haplotype graphs for European and non-European samples were constructed on each 23andMe genotyping platform from a representative sample of genotyped individuals, and then performed out-of-sample phasing of all genotyped individuals against the appropriate graph.

In preparation for imputation, phased chromosomes were split into segments of no more than 10,000 genotyped SNPs, with overlaps of 200 SNPs. SNPs with Hardy–Weinberg equilibrium (HWE) *P*<10^−20^, call rate <95%, or with large allele frequency discrepancies compared with European 1000 Genomes reference data were excluded. Frequency discrepancies were identified by computing a 2 × 2 table of allele counts for European 1000 Genomes samples and 2,000 randomly sampled 23andMe customers with European ancestry, and identifying SNPs with a *χ*^2^ *P*<10^−15^. Each phased segment was imputed against all-ethnicity 1000 Genomes haplotypes (excluding monomorphic and singleton sites) using Minimac2 (ref. 65) using five rounds and 200 states for parameter estimation. The four SNPs reported here had high imputation *r*^2^ values (rs2009262: 0.991; rs3809060: 0.976; rs6991952: 0.999; and rs370763: 0.991)

### Statistical analysis

*GWA analysis*. Analyses in the discovery cohort were conducted using PLINK^66^ v1.07 (http://pngu.mgh.harvard.edu/~purcell/plink) and R^67^ (www.r-project.org). We tested single-marker associations for men and women separately in a logistic regression model adjusted for age and the first 10 ancestry principal components using allele counts for typed SNPs and imputed dosages for the imputed SNPs and a log-additive genetic model. We then conducted meta-analysis of sex-specific results. In the results section, we present top associations that exceeded genome-wide significance (*P*<5 × 10^−8^) at novel loci. We examined the top associations by inspecting the cluster plots, call rates and HWE *P* values of the genotyped SNPs. To detect Hardy–Weinberg deviation due to genotyping error rather than population stratification, the HWE *P* values were calculated based on a subset of the homogeneous non-Hispanic white samples within the interquartile ranges of the first two principal components. The genomic control parameter *λ* was calculated for each analysis to assess inflation due to population stratification. To identify independent signals, we tested the genome-wide SNP associations with inguinal hernia by conditioning on the top SNPs from each of the four independent loci that reached genome-wide significance. Analyses in the replication cohort were conducted in men and women separately with covariates for age and the top five principal components. Results were combined in a fixed effects meta-analysis.

*Narrow-sense heritability due to common alleles*. Using inguinal hernia prevalence estimates among non-Hispanic whites in KPNC along with the previously reported prevalence, we evaluated the ranges of narrow-sense heritability explained by common variants tagged by all SNPs on the Axiom EUR array and separately for the four inguinal hernia risk variants identified in this study. These analyses were carried out as has been previously described, and as implemented in GCTA-REML version 1.2 (http://cnsgenomics.com/software/gcta/reml.html). Briefly, we estimated the variance in case–control status by a linear mixed model of case–control status explained by an additive polygenic variance model of all SNPs as random effects with structure from the estimated realized relationship matrix estimated from the SNP data, and additionally adjusted for the fixed effects of age, sex and first 10 ancestry principal components. These variance estimates were then transformed to the liability scale in the classical liability threshold model via a probit transformation and ascertainment corrected to estimate the SNP heritability, which required specification of the prevalence of the inguinal hernia. To accurately assess the proportion of variance explained, we selected three controls for each case matched on sex and within 3 years of age. When more than three subjects were available, we chose as controls those subjects with minimal genetic distances from the cases, defined as the Euclidean distance of the first two principal components.

*Gene set enrichment analysis. *We conducted gene enrichment set analysis using the MAGENTA software (http://www.broadinstitute.org/mpg/magenta/). To do this, we input a ranked list of our meta-analysis association results. We evaluated the results of the gene set enrichment analysis by nominal *P* value and FDR to control for multiple testing^68^. We considered an FDR<0.05 as significant.

*Identification of potentially regulatory SNPs at inguinal hernia risk loci*. We investigated the regulatory function of non-coding variants using RegulomeDB (http://regulome.stanford.edu)^30^. RegulomeDB synthesizes information from ENCODE and other resources to determine potential regulatory function of specific variants, including those in linkage disequilibrium with associated variants but not themselves tested for association.

*Mouse tissue dissection, qRT-PCR and RNA-seq*. We dissected connective tissue equivalent to human transversalis fascia from the kidney capsule of 12 adult (8–10 weeks old) male CD1 mice (Charles River). This connective tissue is equivalent to human transversalis fascia, in that it is a connective tissue layer in intimate contact with the peritoneum and can be dissected cleanly without contamination from other tissues. Connective tissue from four mice was pooled and RNA isolated using RNeasy Fibrous Tissue Mini Kit (Qiagen). The quality of RNA was examined on a Bioanalyzer 2100 (Agilent) and the RIN values were ≥8.5. For qRT-PCR 200 ng of total RNA was linearly amplified using the qScript cDNA SuperMix (Quanta Biosciences). Reverse transcriptase and PCR conditions were essentially as described using SsoFast EvaGreen (Bio-Rad)^69^. Primer sequences were obtained from the PrimerBank database (Supplementary Table 6)^70^. Reactions were run on a Realplex2 (Eppendorf) thermal cycler. Three samples were analysed in three replicates of each reaction and relative expression levels were calculated by the ΔC_T_ method, normalizing to the housekeeping gene *Hprt*^69^. For RNA-seq, three replicates were generated using 500 ng of total RNA and the TruSeq stranded mRNA kits (Illumina), according to the manufacturer's protocol. Fragment size distribution was assessed using the Bioanalyzer 2100 and the DNA high-sensitivity chip (Agilent). Concentrations of the libraries were measured using the Kapa library quantification kit (Kapa Biosystems). Libraries were multiplexed at a density of three per flow-cell lane and single-end 50 bp reads were obtained by sequencing on a HiSeq 2500 to a depth of at least 34 million reads. Raw sequencing reads were mapped to the mouse genome (mm9) using Tophat2 (doi:10.1186/gb-2013-14-4-r36). Normalized gene expression values, FPKM, were obtained for each replicate using Cufflinks2 (doi:10.1038/nbt.2450).

*Ingenuity pathway analysis. *Causal Network Analysis was carried out using IPA (Qiagen). To narrow down the number of genes for this analysis, we ranked results from the RNA-seq experiment by FPKM value and used an arbitrary cutoff of FPKM 30 (2,059 genes). Of the four genes, two exceeded the cutoff values, *Efemp1* (FPKM: 621, rank: 99) and *Wt1* (FPKM: 35, rank: 1,767), but not *Ebf2* (FPKM: 4.8, rank: 8,723) or *Adamts6* (FPKM: 1.2 rank: 12,467).

## Additional information

**How to cite this article:** Jorgenson, E. *et al.* A genome-wide association study identifies four novel susceptibility loci underlying inguinal hernia. *Nat. Commun.* 6:10130 doi: 10.1038/ncomms10130 (2015).

## Supplementary Material

Supplementary Information Supplementary Figures 1-2, Supplementary Tables 1-6

## Figures and Tables

**Table 1:** SNP associations reaching genome-wide significance in the combined analysis of discovery and replication cohorts.

**SNP**	**Chr.**	**Position**	**Gene**	**Risk allele**	**Discovery**			**Replication**			**Combined**	
					**(5,295 cases, 67,510 controls)**			**(9,701 cases, 82,743 controls)**			**OR (95% CI)**	***P***-**value**
					**RAF**	**OR (95% CI)**	***P***-**value**	**RAF**	**OR (95% CI)**	***P***-**value**		
rs2009262	2	56,012,214	*EFEMP1*	T	0.78	1.23 (1.17–1.30)	3.66 × 10^−15^	0.78	1.10 (1.06–1.15)	3.65 × 10^−06^	1.15 (1.11–1.19)	1.45 × 10^−17^
rs370763	5	64,355,060	*ADAMTS6*	A	0.65	1.14 (1.09–1.19)	9.70 × 10^−09^	0.67	1.06 (1.02–1.09)	3.02 × 10^−03^	1.09 (1.06–1.12)	3.73 × 10^−9^
rs6991952	8	25,707,412	*EBF2*	G	0.43	1.14 (1.10–1.19)	1.17 × 10^−10^	0.43	1.08 (1.05–1.12)	2.04 × 10^−06^	1.11 (1.08–1.14)	6.68 × 10^−15^
rs3809060	11	32,458,807	*WT1*	G	0.62	1.18 (1.13–1.23)	4.69 × 10^−14^	0.63	1.07 (1.03–1.10)	1.69 × 10^−04^	1.11 (1.08–1.14)	3.69 × 10^−14^

Chr., chromosome; CI, confidence interval; RAF, risk allele frequency; SNP, single-nucleotide polymorphism.

**Table 2:** Sex-stratified analysis of direct and indirect inguinal hernia among GERA discovery cohort.

**SNP**	**Inguinal Hernia Type**	**Men**		**Women**		**Combined**					
		**OR (95% CI)**	***P***-**value**	**OR (95% CI)**	***P***-**value**	**OR**_**F**_	***P***_**F**_	**OR**_**R**_	***P***_**R**_	**I**^**2**^	***P***_**Het**_
rs2009262	Direct	1.25 (1.16–1.36)	2.31 × 10^−8^	1.42 (1.04–1.94)	0.03	1.26 (1.17–1.36)	2.81 × 10^−9^	1.26 (1.17–1.36)	2.81 × 10^−9^	0	0.46
	Indirect	1.21 (1.13–1.31)	4.48 × 10^−7^	1.51 (1.18–1.93)	0.001	1.24 (1.15–1.33)	8.48 × 10^−9^	1.31 (1.07–1.60)	0.009	62.4	0.1
rs370763	Direct	1.14 (1.06–1.22)	1.60 × 10^−4^	1.22 (0.94–1.58)	0.133	1.14 (1.07–1.22)	6.38 × 10^−5^	1.14 (1.07–1.22)	6.38 × 10^−5^	0	0.65
	Indirect	1.15 (1.08–1.23)	2.86 × 10^−5^	1.10 (0.91–1.34)	0.332	1.14 (1.08–1.22)	2.28 × 10^−5^	1.14 (1.08–1.22)	2.28 × 10^−5^	0	0.66
rs6991952	Direct	1.21 (1.14–1.29)	8.95 × 10^−10^	1.03 (0.81–1.30)	0.814	1.20 (1.13–1.28)	2.38 × 10^−9^	1.16 (1.01–1.34)	0.044	46	0.17
	Indirect	1.14 (1.08–1.21)	1.17 × 10^−5^	1.07 (0.89–1.28)	0.461	1.14 (1.07–1.20)	1.20 × 10^−5^	1.14 (1.07–1.20)	1.20 × 10^−5^	0	0.46
rs3809060	Direct	1.21 (1.13–1.29)	2.71 × 10^−8^	1.44 (1.11–1.86)	0.006	1.22 (1.14–1.30)	1.55 × 10^−9^	1.26 (1.09–1.46)	0.003	44.7	0.18
	Indirect	1.17 (1.09–1.24)	2.16 × 10^−6^	1.55 (1.26–1.89)	2.41 × 10^−5^	1.20 (1.13–1.27)	8.14 × 10^−9^	1.32 (1.00–1.74)	0.051	85.9	0.01

CI, confidence interval; GERA, Genetic Epidemiology Research in Adult Health and Aging; I^2^, heterogeneity index; OR_F_, odds ratio from fixed effects model; OR_R_, odds ratio from random effects model; *P*_Het_, *P* value for heterogeneity from Cochran's Q test; *P*_F_, *P* value from fixed effects model; *P*_R_, *P* value from random effects model; SNP, single-nucleotide polymorphism.
