# Supplementary material for: A genome-wide association study identifies four novel susceptibility loci underlying inguinal hernia
Source: Nat Commun. 2015 Dec 21;6:10130. doi: 10.1038/ncomms10130 (PMC4703831; doi:10.1038/ncomms10130)
Supplement: Supplementary Information — Supplementary Figures 1-2, Supplementary Tables 1-6 [file ncomms10130-s1.pdf]

Supplementary Figures

Supplementary Figure 1: (A-D) Locus Zoom plots of the top four loci associated with inguinal hernia in the GERA discovery cohort. Plots show association results of SNPs in the GWAS of the GERA cohort samples and recombination rates. Each circle represents the $-\log_{10}$ *P* value (y axes) and chromosomal position (x axes) of SNPs tested for association. The top associated SNP in each region is identified by its rsID. The color of each circle reflects the extent of LD with the top SNP. Physical positions are based on NCBI build 37 of the human genome. The relative position of genes and transcripts are displayed below the association plot.

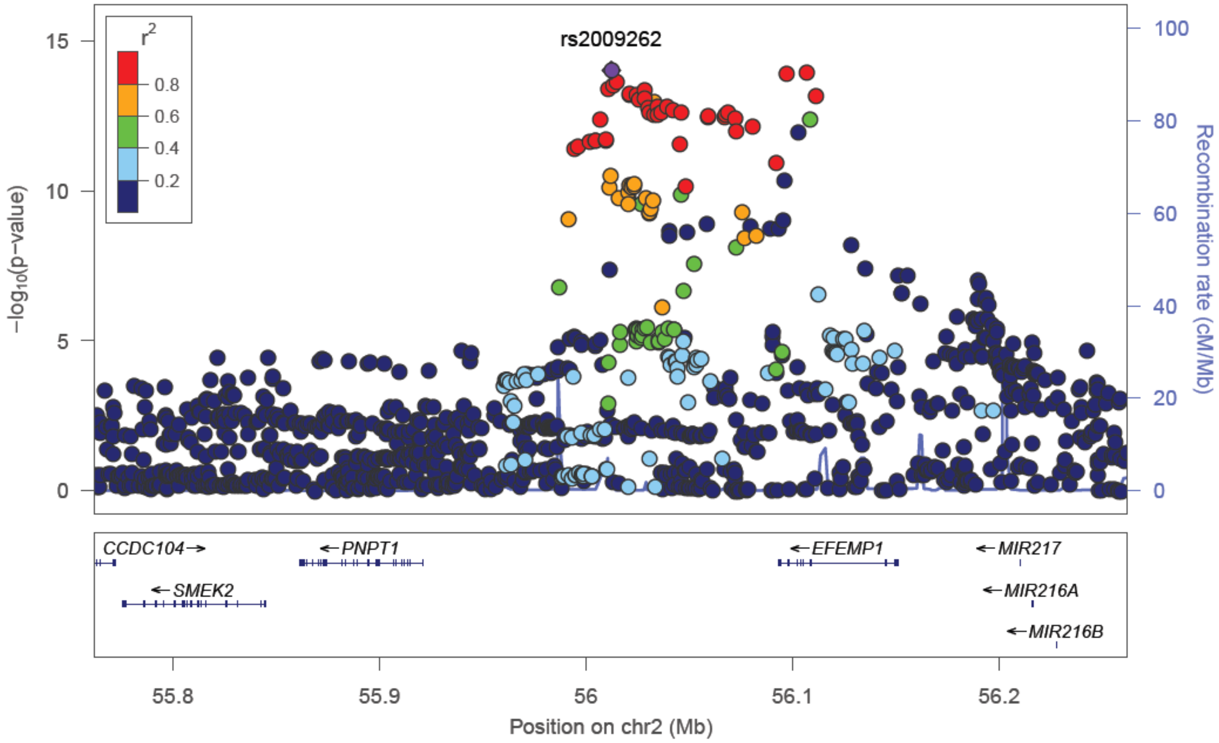

A. Chromosome 2

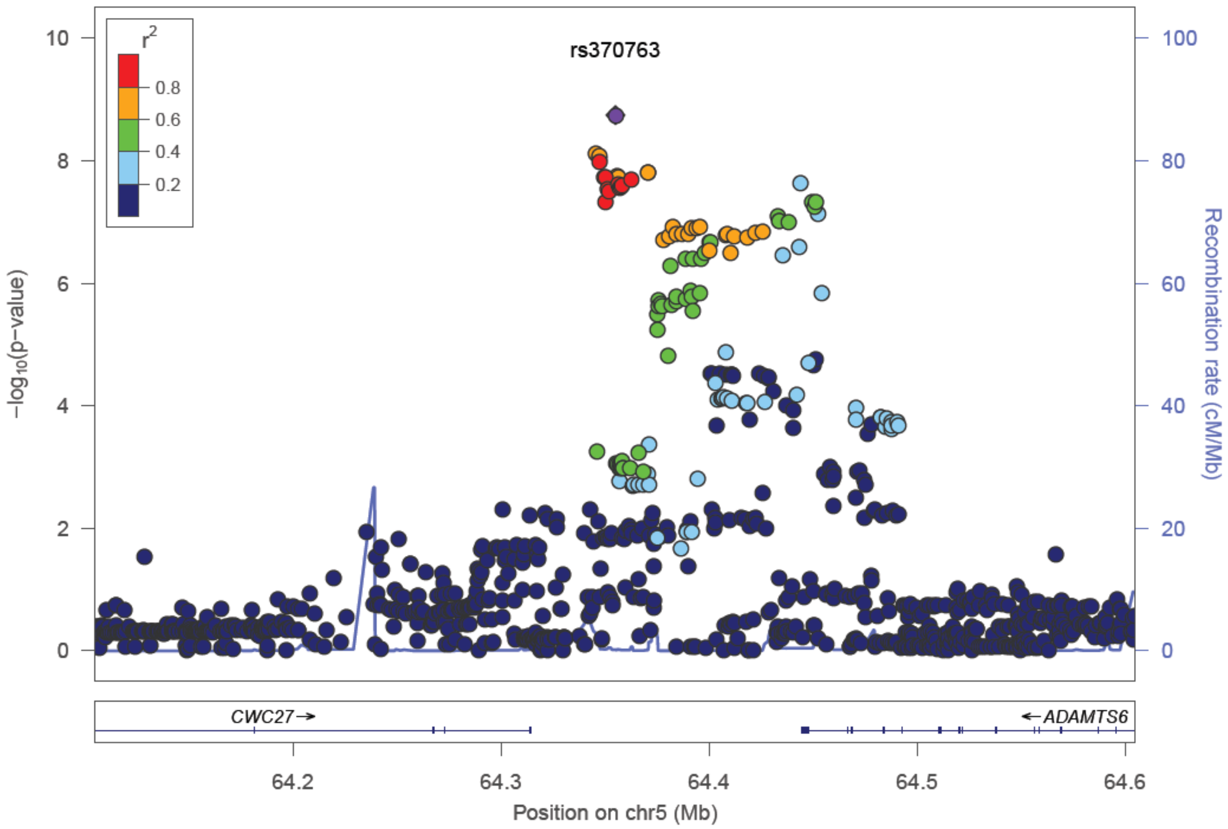

B. Chromosome 5

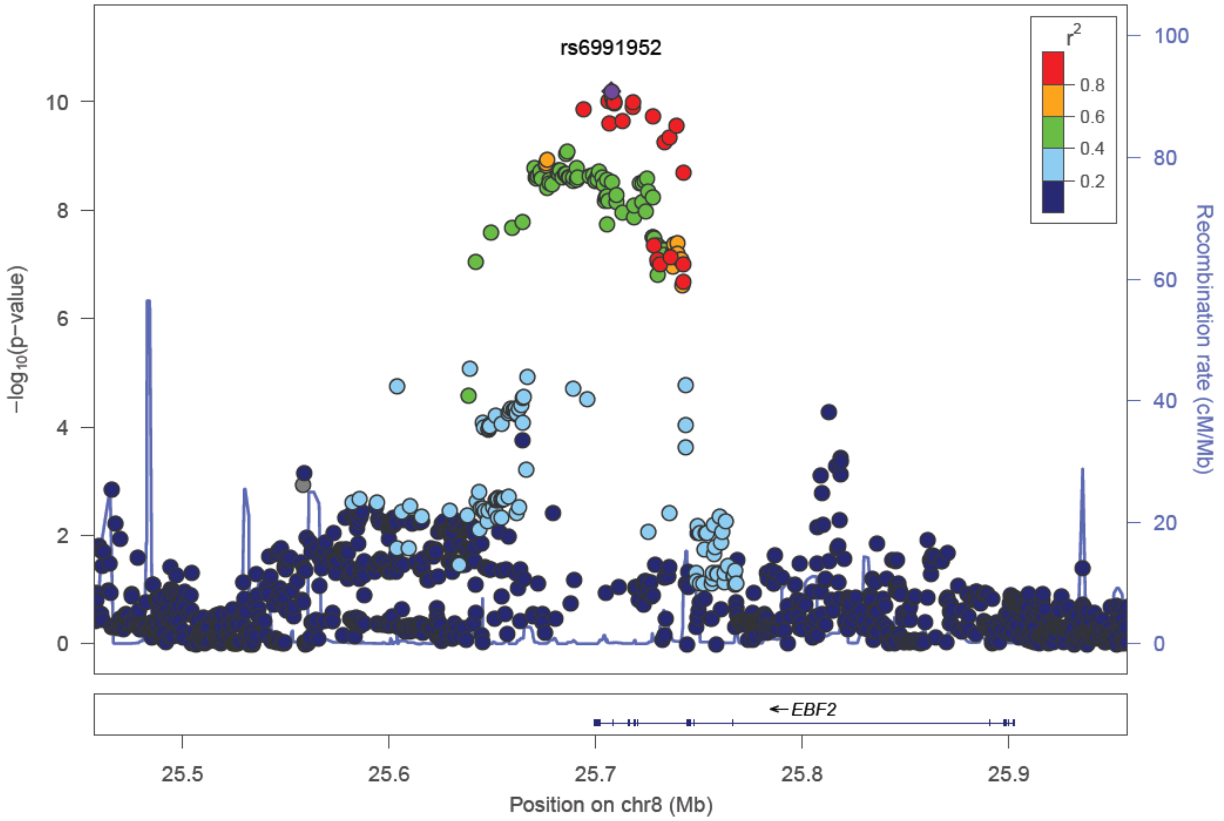

C. Chromosome 8

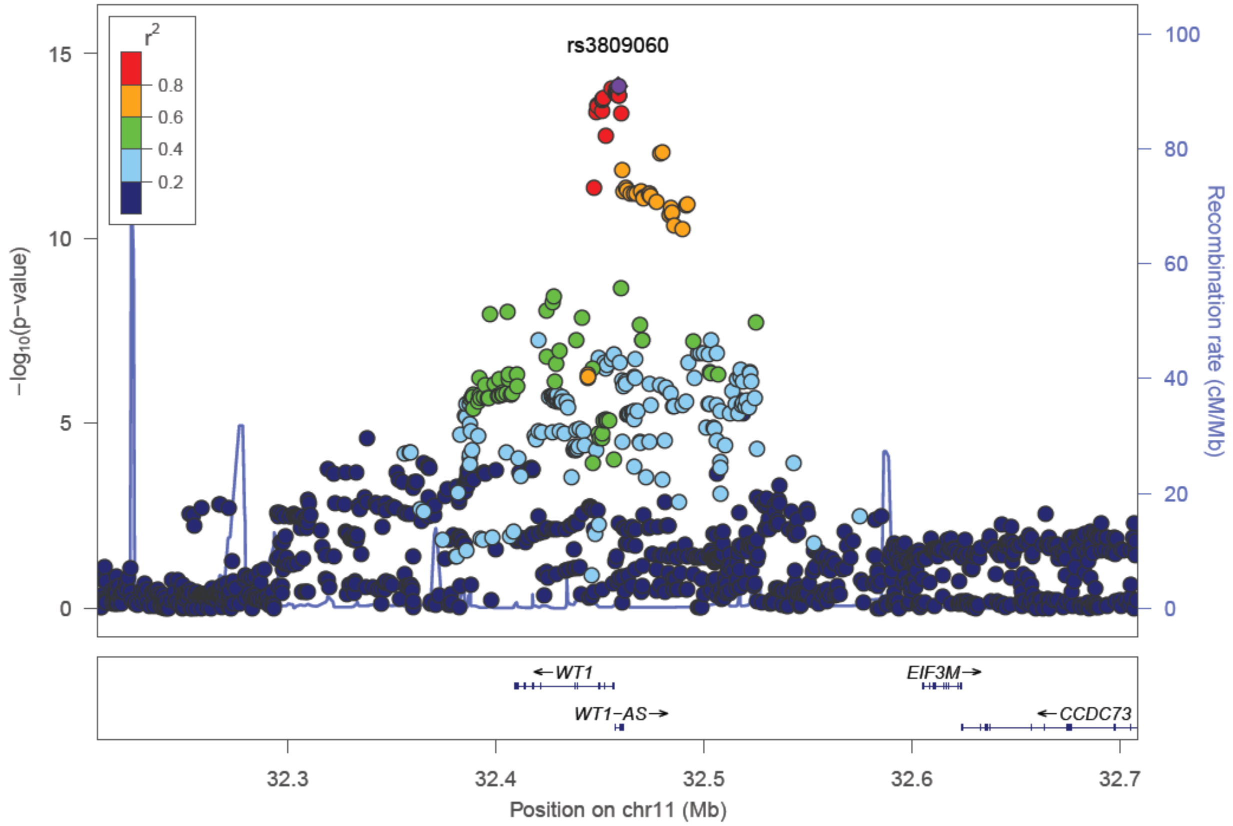

D. Chromosome 11

Supplementary Figure 2: (A-H) Locus Zoom plots of the top four loci associated with inguinal hernia in GERA cohort men, for direct and indirect inguinal hernia. Plots show association results of SNPs in the GWAS of the GERA cohort samples and recombination rates. Each circle represents the -$\log_{10}$ *P* value (y axes) and chromosomal position (x axes) of SNPs tested for association. The top associated SNP in each region is identified by its rsID. The color of each circle reflects the extent of LD with the top SNP. Physical positions are based on NCBI build 37 of the human genome. The relative position of genes and transcripts are displayed below the association plot.

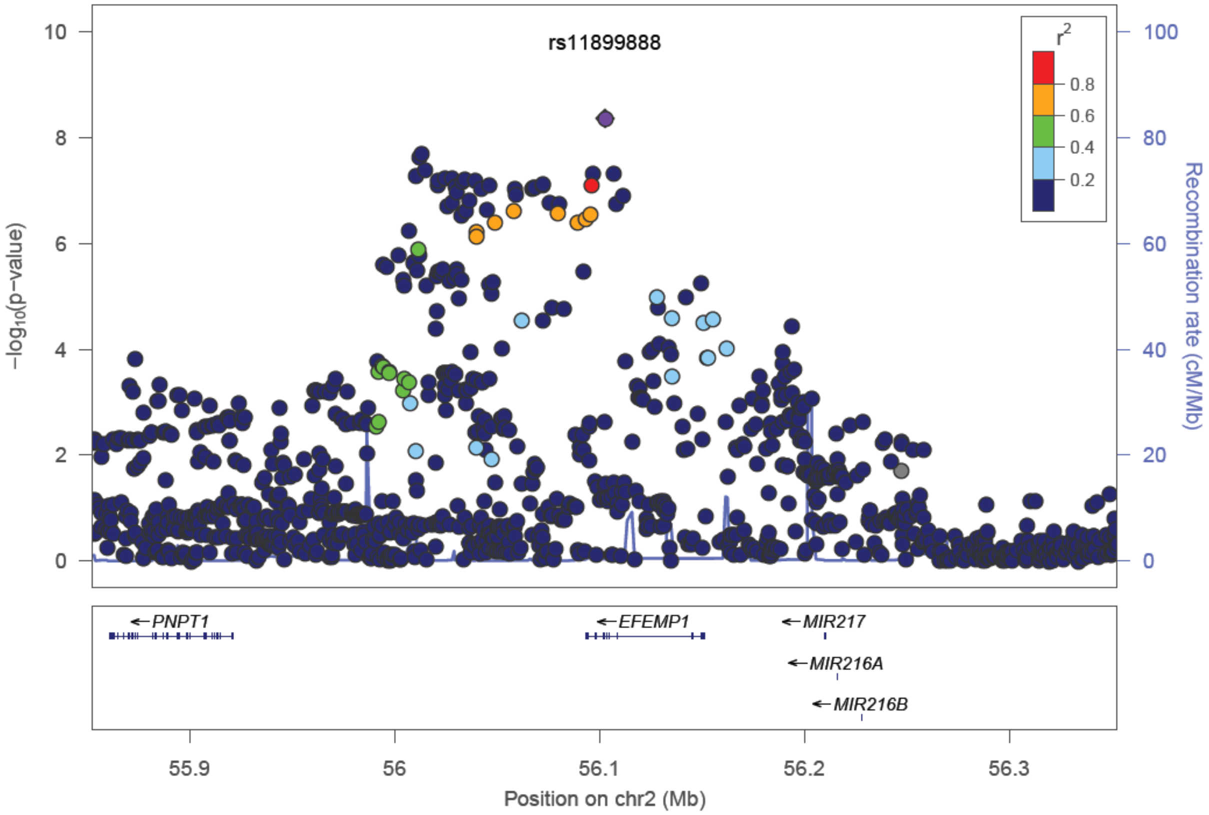

A. Chromosome 2 Direct Inguinal Hernia in GERA Men

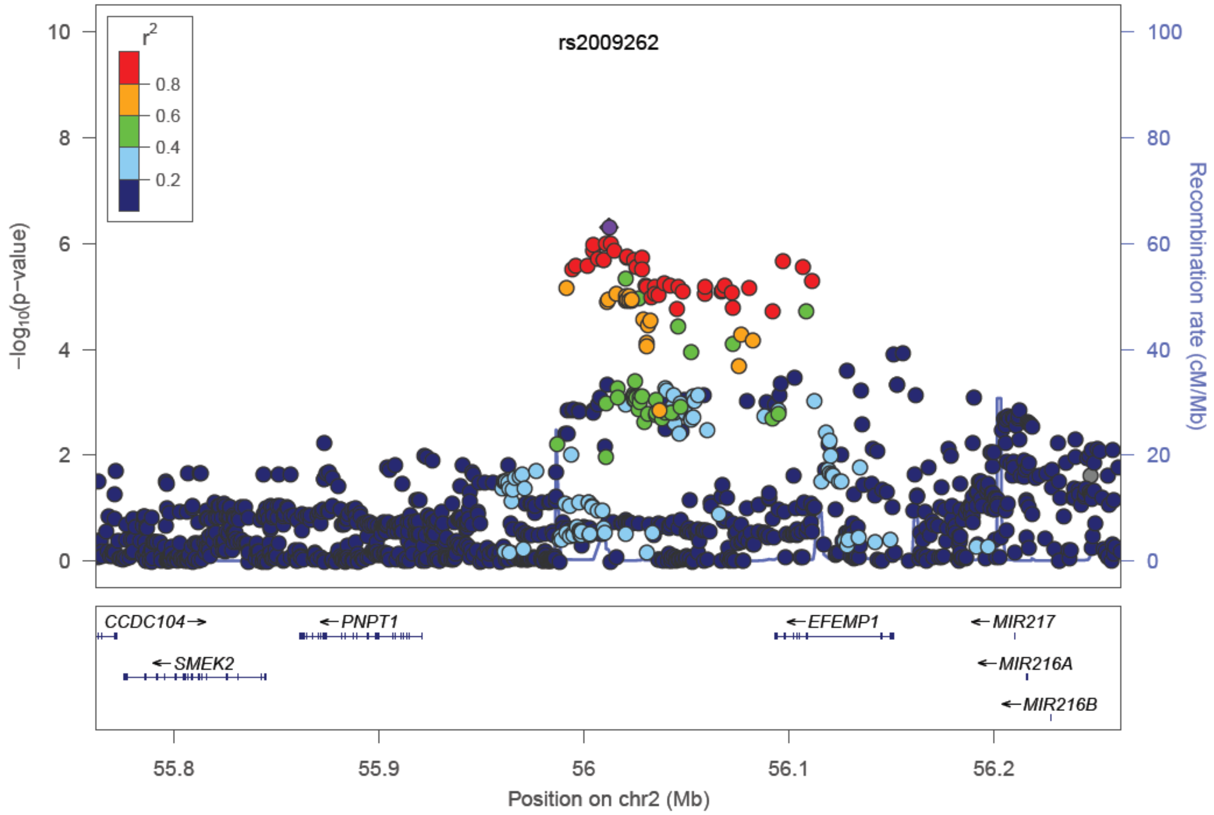

B. Chromosome 2 Indirect Inguinal Hernia in GERA Men

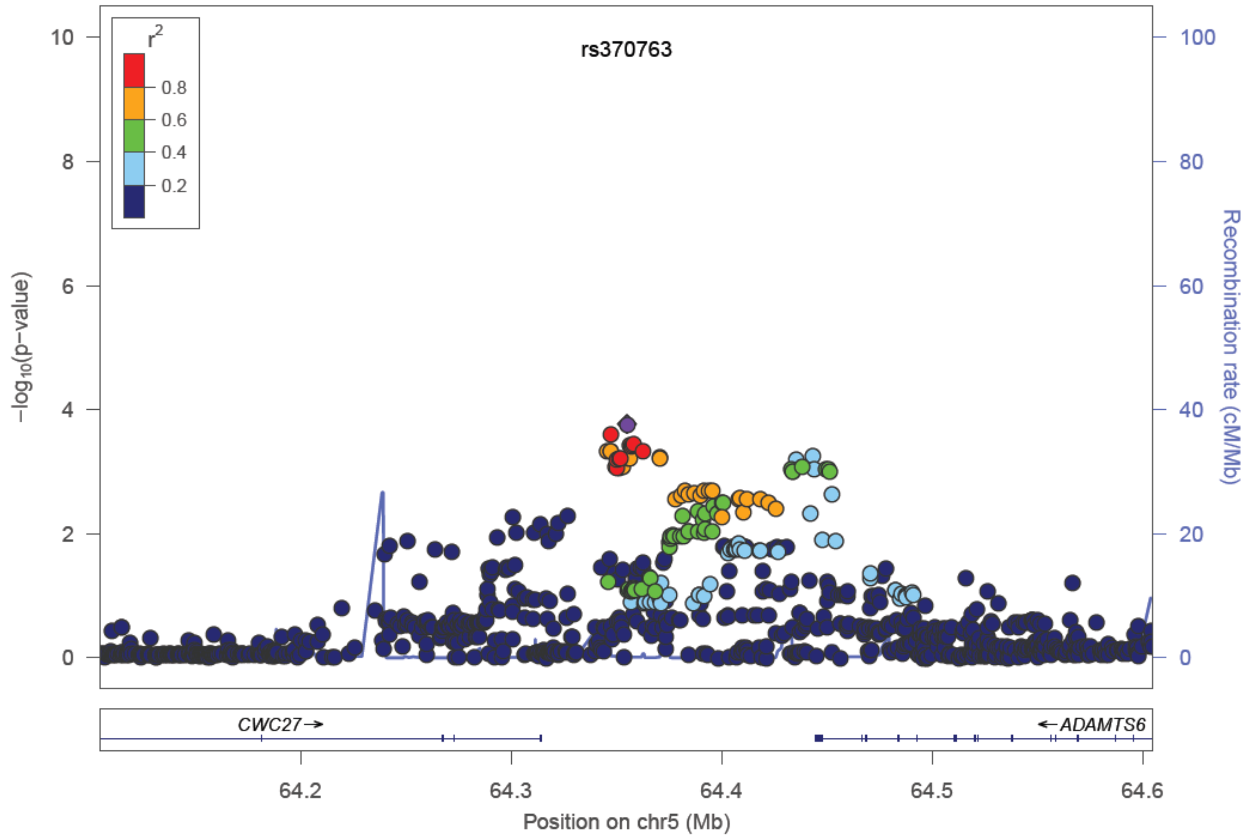

C. Chromosome 5 Direct Inguinal Hernia in GERA Men

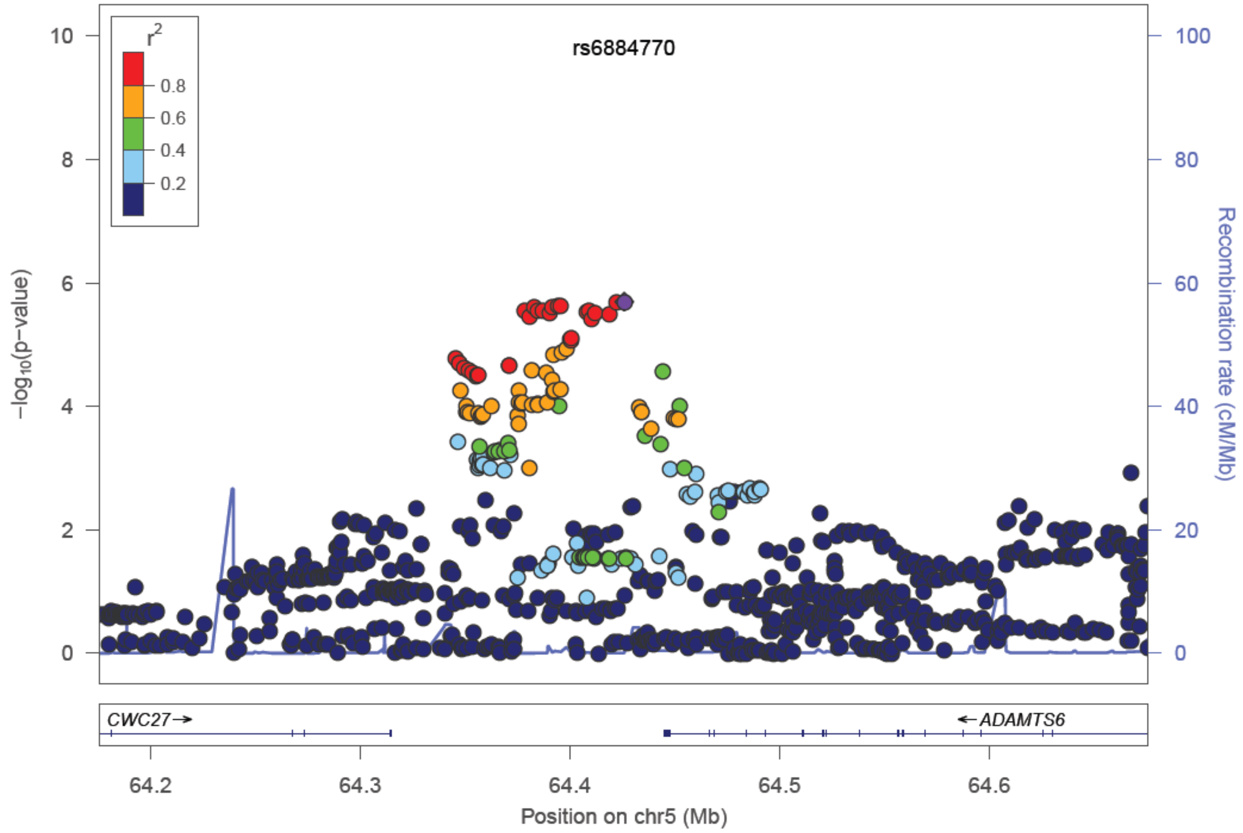

D. Chromosome 5 Indirect Inguinal Hernia in GERA Men

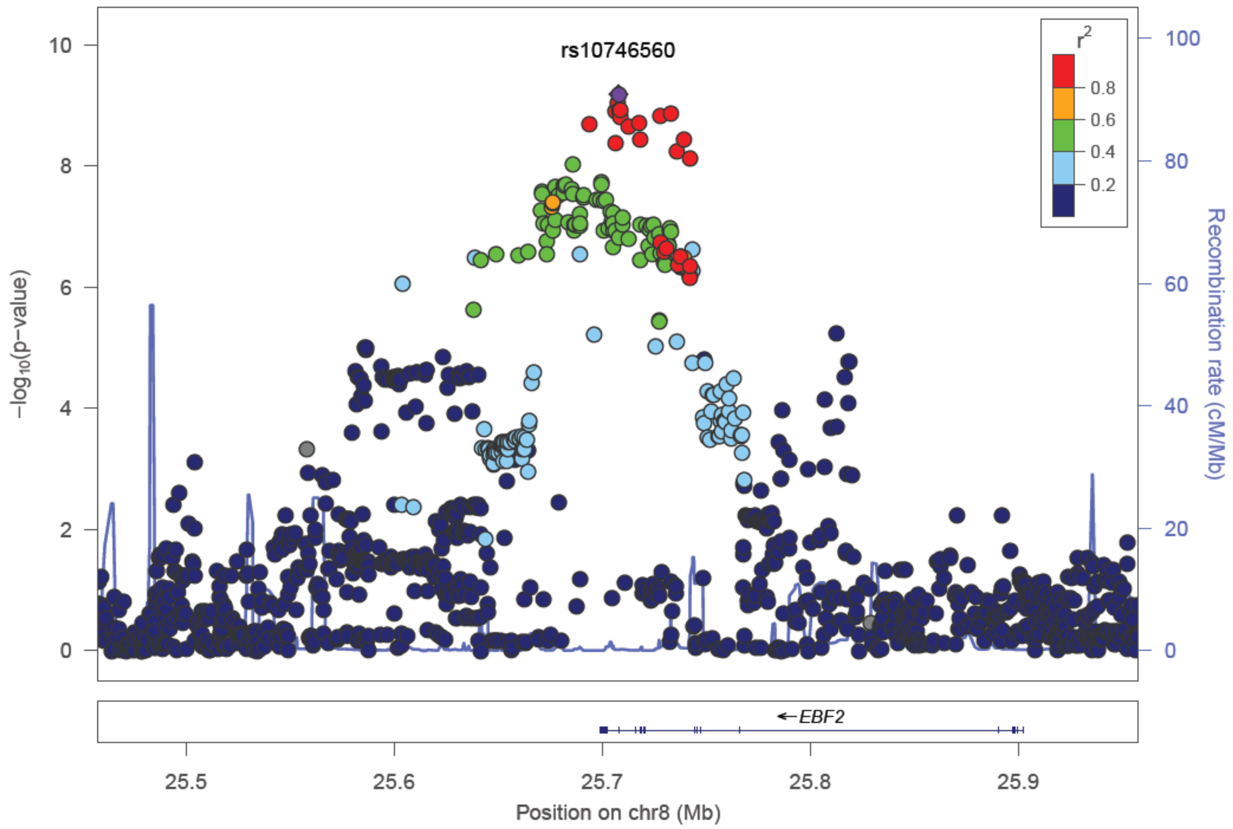

E. Chromosome 8 Direct Inguinal Hernia in GERA Men

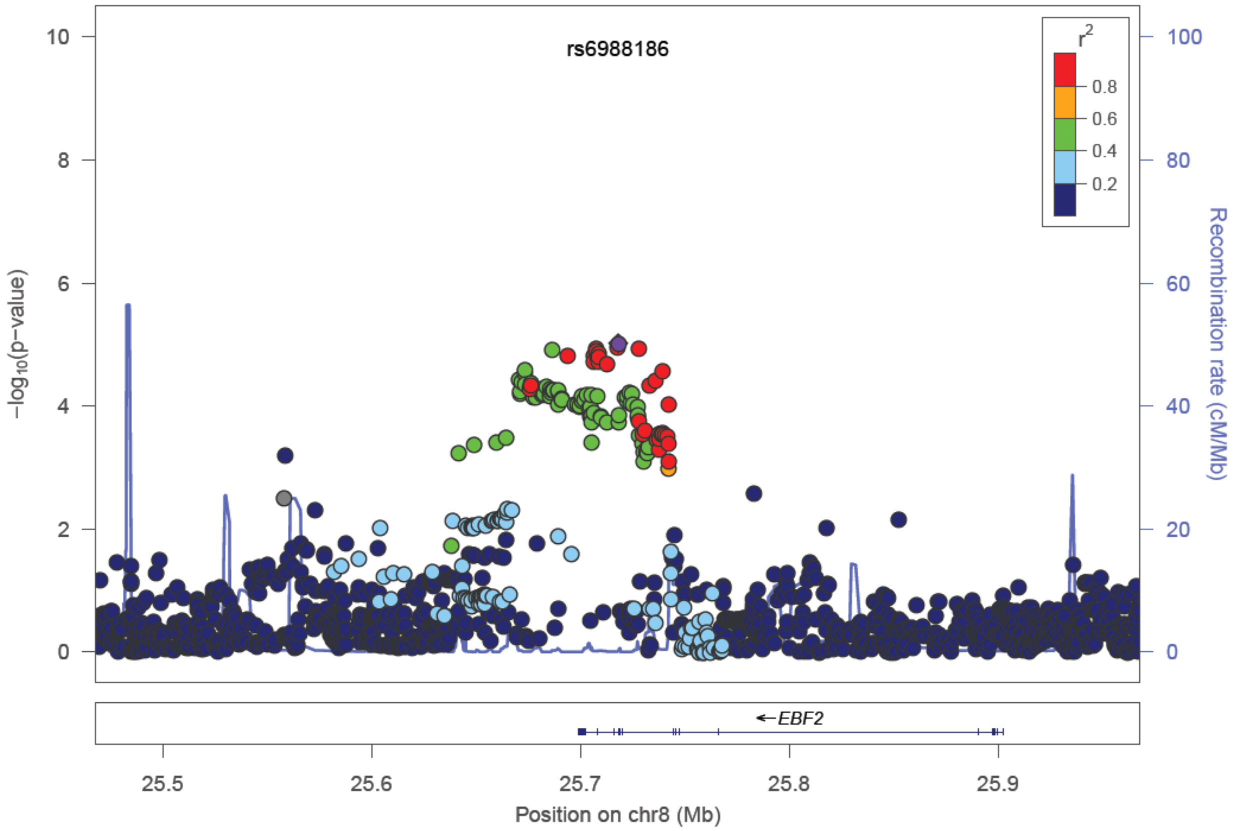

F. Chromosome 8 Indirect Inguinal Hernia in GERA Men

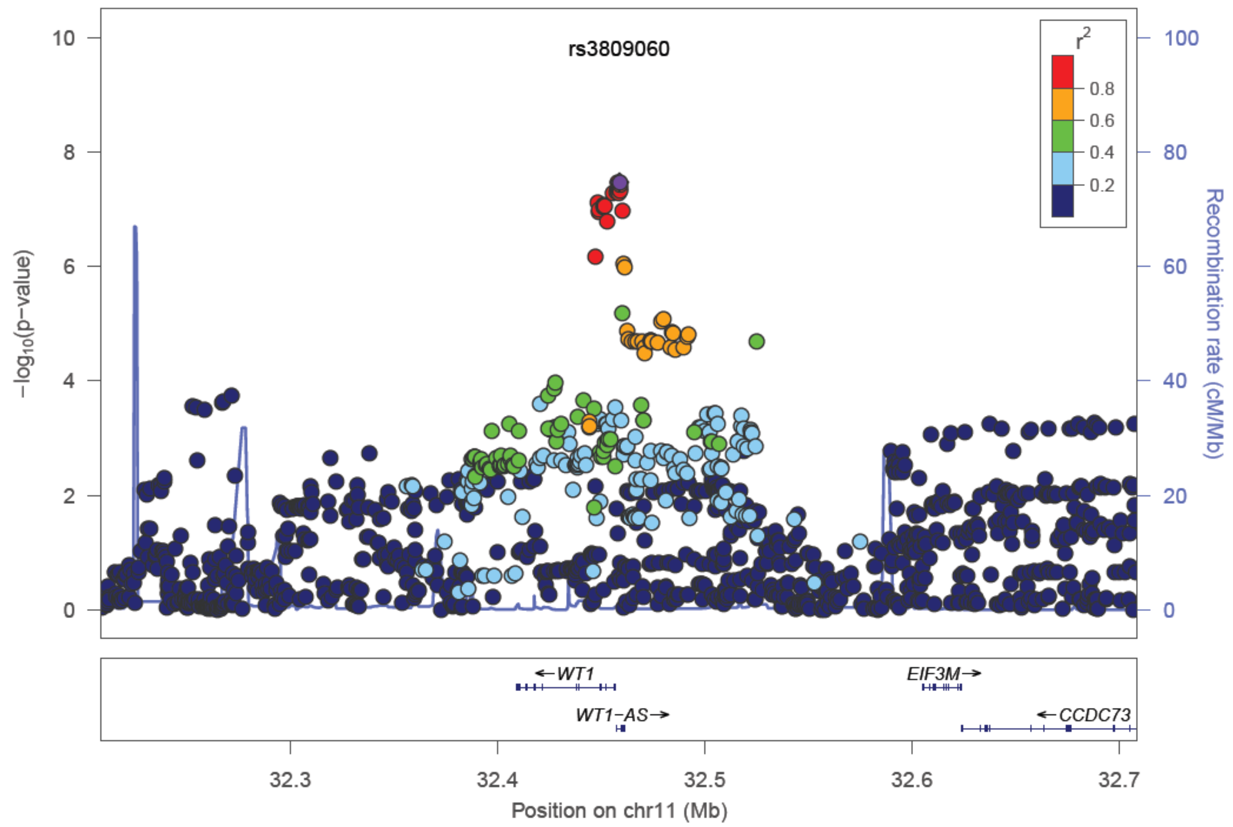

G. Chromosome 11 Direct Inguinal Hernia in GERA Men

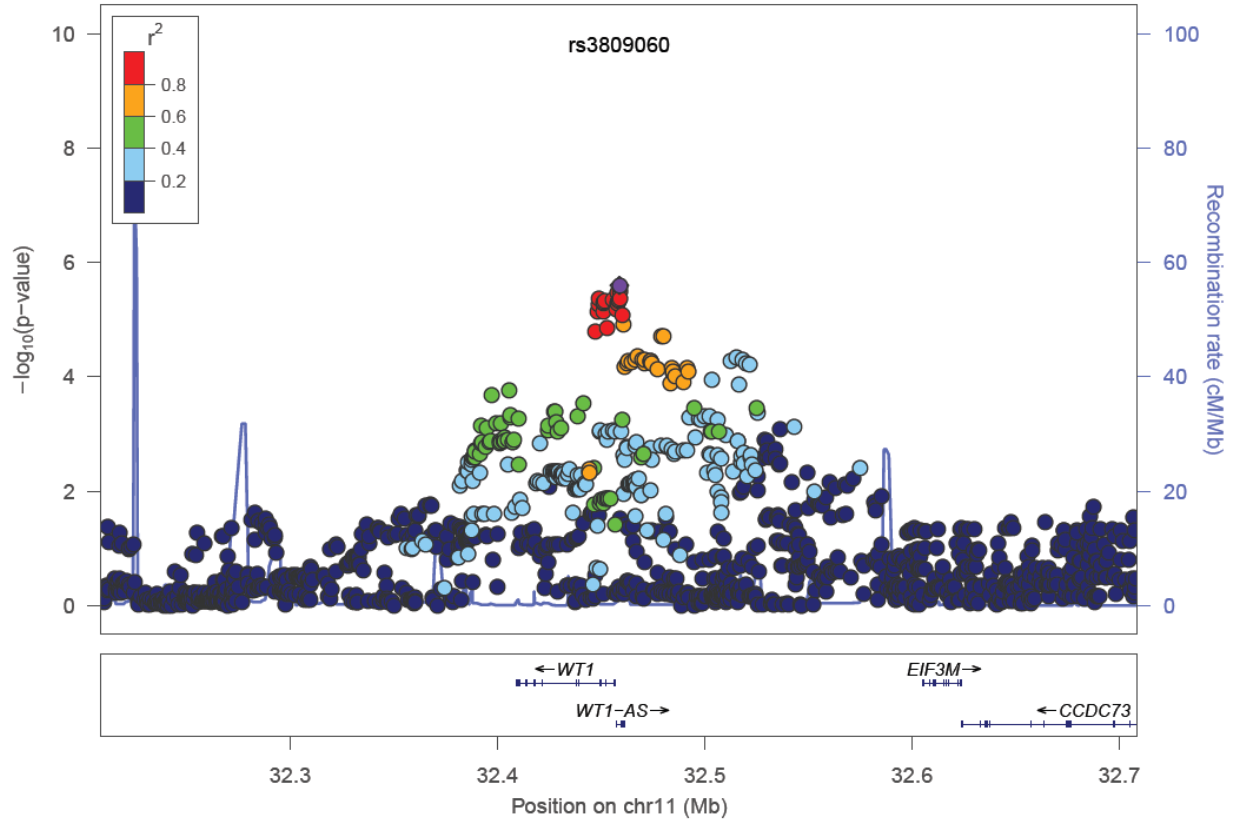

H. Chromosome 11 Indirect Inguinal Hernia in GERA Men

Supplementary Tables

Supplementary Table 1: Characteristics of GERA discovery cohort subjects

	Inguinal Hernia (n=5,295)						Controls (n=67,510)	
	All (n=5,295)		Direct (n=2,335)		Indirect (n=2,647)			
	N	%	N	%	N	%	N	%
Men	4,746	89.6%	2,193	93.9%	2,408	91.0%	25,011	37.1%
Women	549	10.4%	142	6.1%	239	9.0%	42,499	63.0%
	Mean	SD	Mean	SD	Mean	SD	Mean	SD
Age At Specimen Collection	70.1	10.6	69.7	10.3	69.8	10.5	62.6	13.4

Supplementary Table 2: Characteristics of the 23andMe replication cohort subjects

		Hernia repair cases (n=9,701)		Controls (n=82,743)	
Men		6,812	70.2%	41,611	50.2%
Age					
	<30	246	3.6%	5,147	12.4%
	31-45	1,087	16.0%	14,109	33.9%
	46-60	1,605	23.6%	10,259	24.7%
	>60	3,874	56.9%	12,096	29.1%
Women		1,889	29.8%	41,132	49.8%
Age					
	<30	63	3.3%	4,252	10.3%
	31-45	317	16.8%	11,099	27.0%
	46-60	603	31.9%	11,431	27.8%
	>60	906	48.0%	14,350	34.9%

Supplementary Table 3: Gene sets enriched for association signals in the GSEA (FDR < 0.05)

Gene Set	Database	Gene Set Size	Median Gene Size	p	FDR
JAK Stat Signaling	Ingenuity	10	53	0.0011	0.0125
Leukocyte Extravasation Signaling	Ingenuity	47	39	0.0002	0.0149
Actin Cytoskeleton Signaling	Ingenuity	28	80	0.0018	0.0273
Glycosaminoglycan Biosynthesis Chondroitin Sulfate	KEGG	22	40	0.0006	0.0276

Supplementary Table 4: SNPs classified as likely to affect binding according to RegulomeDB in the four inguinal hernia risk loci

SNP	Chromosome	Start Position	End Position	RegulomeDB Score
rs11888023	2	56,006,984	56,006985	2b
rs6739641	2	56,020,961	56,020962	2b
rs3791679	2	56,096,891	56,096892	2b
rs447583	5	64,357,582	64,357583	2b
rs264735	5	64,362,410	64,362411	2b
rs6449773	5	64,438,515	64,438516	2b
rs4568629	8	25,729,751	25,729752	2b
rs7925851	11	32,396,171	32,396172	2b
rs10835894	11	32,401,413	32,401414	2b
rs11031762	11	32,407,853	32,407854	2b
rs5030178	11	32,448,568	32,448569	2b
rs11031779	11	32,452,887	32,452888	2b
rs1799925	11	32,456,561	32,456562	2b
rs2301251	11	32,457,984	32,457985	2b

Supplementary Table 5: Diagnosis and procedure codes

Hernia Type	ICD9 Diagnosis Codes	ICD9 Procedure Codes	CPT4 Codes
Inguinal Hernia	550.x	17.x, 53.0x, 53.1x	49491, 49492, 49495, 49496, 49500, 49501, 49505, 49507, 49520, 49521, 49525, 49650, 49651, 49659
Direct Inguinal		17.11, 17.21, 17.23, 53.01, 53.03, 53.11, 53.14, 53.13, 53.16, 53.17	
Indirect Inguinal		17.12, 17.22, 17.23, 53.02, 53.04, 53.12, 53.15, 53.13, 53.16, 53.17	

Supplementary Table 6: Primer sequences for qRT-PCR

Gene	Primer Sequence
Efemp1_F	GCGCTGGTCAAGTCACAGTA
Efemp1_R	AAGCATCTGGGACAATGTCAC
Wt1_F	GAGAGCCAGCCTACCATCC
Wt1_R	GGGTCCTCGTGTTTGAAGGAA
Ebf2_F	GGGATTCAAGATACGCTAGGAAG
Ebf2_R	GGAGGTTGCTTTTCAAAATGGG
Adamts6_F	TGGTGGCTGAATAAGACATCATC
Adamts6_R	GGAGTCACGATAAAGTTTGGCAA
Col12a1_F	TTGCAGCTAGTACCACTGAAAC
Col12a1_R	CCCTGGCTTTGTAGGACCAC
Oct4_F	AGCGATCAAGCAGCGACTAT
Oct4_R	TAGCCTGGGGTACCAAAATG
